# Individual-specific changes in circadian rest-activity rhythm and sleep in symptom-free patients tapering their antidepressant medication

**DOI:** 10.1192/j.eurpsy.2022.1747

**Published:** 2022-09-01

**Authors:** O. Minaeva, E. Schat, E. Ceulemans, Y. Kunkels, A. Smit, M. Wichers, S. Booij, H. Riese

**Affiliations:** 1 University of Groningen, University Medical Center Groningen, Department Of Psychiatry, Interdisciplinary Center Psychopathology And Emotion Regulation, Groningen, Netherlands; 2 KU Leuven, Faculty Of Psychology And Educational Sciences, Leuven, Belgium; 3 Lentis, Center For Integrative Psychiatry, Groningen, Netherlands

**Keywords:** individual models, circadian rhythm, Depression, sleep

## Abstract

**Introduction:**

Group-level studies showed cross-sectional and prospective between-person associations between circadian rest-activity rhythms (RAR), physical activity (PA), sleep, and depressive symptoms. However, whether these associations replicate at the within-person level remains unclear. Therefore, it is clinically relevant to investigate these associations within persons and study whether changes in depressive symptoms are related to changes in circadian rhythm and sleep variables.

**Objectives:**

To identify changes in circadian rhythm elements in proximity to a transition in depressive symptoms, whether changes are less frequent in individuals without compared to those with transitions, and whether there are individual differences in the direction of change of circadian rhythm variables.

**Methods:**

Data of remitted individuals tapering antidepressants were used: 12 with and 14 without a transition in depressive symptoms. RAR, PA, and sleep variables were calculated as predictors from four months of actigraphy data. Transitions in depressive symptoms were based on weekly SCL-90 scores and evaluation interviews. Kernel Change Point analyses were used to detect change points (CPs) and CP timing in circadian rhythm variables for each individual separately.

**Results:**

In 67% of individuals with depressive symptoms transitions, CPs were identified in proximity to symptom transitions. CPs were detected less frequently in the no-transition group with 7 CPs in 14 individuals, compared to transition groups with 10 CPs in 12 individuals. For several RAR and sleep variables, consistent changes were detected in expected directions.

**Conclusions:**

Circadian rhythm variables provide potentially clinically relevant information although their patterns around transitions are highly person-specific. Future research is needed to disentangle which variables are predictive for which patients.

**Disclosure:**

No significant relationships.

